# Activation of TRPV1 Contributes to Recurrent Febrile Seizures *via* Inhibiting the Microglial M2 Phenotype in the Immature Brain

**DOI:** 10.3389/fncel.2019.00442

**Published:** 2019-10-11

**Authors:** Weilin Kong, Xin Wang, Xingliang Yang, Wenxian Huang, Song Han, Jun Yin, Wanhong Liu, Xiaohua He, Biwen Peng

**Affiliations:** ^1^Department of Physiology, Hubei Provincial Key Laboratory of Developmentally Originated Disease, School of Basic Medical Sciences, Wuhan University, Wuhan, China; ^2^Department of Pathology, Renmin Hospital of Wuhan University, Wuhan, China; ^3^Department of Pathophysiology, School of Basic Medical Sciences, Wuhan University, Wuhan, China; ^4^Department of Immunology, School of Basic Medical Sciences, Wuhan University, Wuhan, China

**Keywords:** TRPV1, recurrent febrile seizure, microglia, TGF-β1, TLR4

## Abstract

Transient receptor potential vanilloid type 1 (TRPV1) is a nonselective cation channel implicated in the nervous system as a key component of several inflammatory diseases. A massive amount of evidence has demonstrated that TRPV1 is extensively expressed in the central nervous system (CNS) and there might be a close relationship between TRPV1 and neuroinflammation, which is a crucial pathogenic factor in seizure generation, although it’s signaling mechanism has been less well characterized. Herein, we identified that TRPV1 is functionally expressed in the primary cultured mouse microglia and the membrane expression of TRPV1 is upregulated in rFS mice brain and specifically in activated microglia. Stimulation of TRPV1 promoted microglia activation and indirectly enhanced seizure susceptibility by inhibiting the neuroprotective effects of microglial transforming growth factor-beta1 (TGF-β1) *via* interaction with Toll-like receptor 4 (TLR4) in mice. Conversely, genetic deletion of TRPV1 alleviated hyperthermia or LPS-induced abnormal microglial activation and restored a balanced inflammatory microenvironment in the brain. Taken together, these findings show that microglial TRPV1, as a potential pro-inflammatory mediator, and participate in neuroinflammatory response, which will provide a novel therapeutic strategy for controlling the neuroinflammation-induced seizure.

## Introduction

Transient receptor potential vanilloid type 1 (TRPV1) is a well-characterized, ligand-gated temperature-sensitive nonselective cationic channel that is activated by a wide variety of exogenous and endogenous factors, such as hyperthermia (>43°C), capsaicin and certain biotoxins (Caterina et al., 1997; Bohlen et al., 2010). TRPV1 behaves as a molecular integrator of hyperthermia and chemical stimuli in the peripheral nervous system (Kim et al., 2012, 2014). However, neurobehavioral studies have recently demonstrated that TRPV1 is broadly expressed in the brain (Hurtado-Zavala et al., 2017; Kong et al., 2017; Marrone et al., 2017) and critically implicated in neurological and mental disorders, such as epilepsy/seizure (Sun et al., 2013; Cho et al., 2018), anxiety, and neurodegenerative diseases (Edwards, 2014). Studies from our laboratory and another laboratory discovered that TRPV1 does not merely aggravate severe seizure behavior but also enhances the susceptibility of pentylenetetrazol-induced seizure in mice after rFSs (Kong et al., 2014; Huang et al., 2015; Jia et al., 2015). However, the functional significance and molecular mechanism of TRPV1 in neuroinflammation-induced seizure remains largely vague.

It is generally considered that TRPV1 activation directly promotes neuronal synaptic transmission and neurogenesis (Saffarzadeh et al., 2015, 2016). In addition, mounting evidence suggests that TRPV1 modulates the delicate interactions between microglia and neurons by which microglia can regulate neuronal activities (Eyo et al., 2017; Marrone et al., 2017). Recent work has shown that TRPV1 is functionally expressed in a large proportion of microglia (Hassan et al., 2014; Miyake et al., 2015; Marrone et al., 2017) and modulates excitatory neurotransmission from microglia (Pascual et al., 2012; Marrone et al., 2017). Aside from excitatory neurotransmission, neuroinflammation associated with the microglial activation is characteristic of epileptic brains (Shapiro et al., 2008). Our previous work not only demonstrated that genetic ablation of TRPV1 function reduces the pro-inflammatory cytokine level (IL-1β, IL-6, TNF, and HMGB1) in rFS mice but also confirmed that overexpression of TRPV1 increases the pro-inflammatory cytokines and decreases the anti-inflammatory cytokines in BV2 microglia (Huang et al., 2015). Actually, TRPV1 is overexpressed in temporal lobe epilepsy (TLE) patients and rFS mice (Sun et al., 2013; Huang et al., 2015). Once activated, TRPV1 mediates a series of transformations ranging from microglial cell death to inflammatory mediators (Kim et al., 2006; Hassan et al., 2014; Miyake et al., 2015; Marrone et al., 2017) under different stimulus conditions.

Moreover, sustained microglial activation can contribute to the generation and recurrence of seizures (Xanthos and Sandkuhler, 2014; Eyo et al., 2017). Therefore, these results indicate that microglial TRPV1 activation is a trigger and detector of neuroinflammation in rFS mice and TLE patients.

Microglial activation is often categorized as either classical (the M1 phenotype) or alternative (the M2 phenotype), following the cue from macrophage studies (Boche et al., 2013; Benson et al., 2015; Hu et al., 2015; Eyo et al., 2017). The M1 phenotype mainly induces neuroinflammation and neurotoxicity (Colonna and Butovsky, 2017), whereas the M2 phenotype mainly releases prosurvival and neuroprotective factors (Hu et al., 2015; Colonna and Butovsky, 2017). Activation of microglial TRPV1 promotes the release of excitotoxicity pro-inflammatory molecules and maintains persistent neuroinflammation, which is a critical etiology of epilepsy/seizures (Robel and Sontheimer, 2016; Eyo et al., 2017; Marrone et al., 2017). Importantly, activated microglia also release multiple anti-inflammatory mediators, including transforming growth factor-beta1 (TGF-β1; Morgan et al., 1993; Benson et al., 2015), which can convert the polarization states of microglia from the M1 phenotype to the M2 phenotype (Boche et al., 2013). However, it is not known whether TRPV1 regulated microglia phenotype changes contribute to seizures.

In the present study, we aimed to explore the roles and underlying molecular mechanism of TRPV1 in the persistent activation of microglia promoting neuroinflammation-induced seizures. The results indicated that TRPV1 contributes to the development of rFSs by inhibiting the M2 microglial activation through TGF-β1 signaling, which as a complementary pathway for M1 microglial activation to promote seizure.

## Materials and Methods

### Animals

All animal care and experimental procedures were approved by the Institutional Animal Care and Use Committee of Wuhan University Medical School. C57BL/6 wild-type (WT) mice were approved by the Hubei Province Center for Animal Experiments, and B6.129X1-Trpv1^tm1Jul/J^ mice (TRPV1^−/−^, KO) were approved by the Nanjing Biomedical Research Institute. All mice were grouped randomly and kept with their mothers under a 12-h light–dark cycle at 25 ± 1°C and a relative humidity of 60~80%, with food and water available *ad libitum* in the animal biosafety level III laboratory (ABSL-III) of Wuhan University. The specific numbers and groups of animals used in each experiment are detailed in the relevant sections of the “Results.”

### Hyperthermia-Induced Seizure

The rFS model with a total of four seizures has been described previously (Kong et al., 2014; Warner et al., 2017). Briefly, on postnatal day 14 (P14), the mice were subcutaneously injected with 0.9% saline as a prevention against dehydration (10 ml/kg body weight) and then were exposed to a preheated glass container in which hyperthermia was induced using a regulated warm air stream with a temperature of 43 ± 0.5°C from an adjustable incubator at the beginning of the experimental febrile seizure for at least 30 min. If the mice had generalized tonic-clonic seizures, they were transferred to a cool surface to recover and return to normothermia, and then were returned back to their mothers for feeding. A total of 48 mice (P14) were used to induce rFS models, of which 30 were WT mice, including four mice mortalities, two mice non-seizure; 18 TRPV1^−/−^ mice were used for inducing rFS models, including four mice mortalities, two mice non-seizure. Seizure latency, duration and seizure grade were presented in Table 1.

**Table 1:** Seizure latency, duration and seizure grade in C57BL/6 WT and TRPV1^−/−^ mice following repetitive exposure to hyperthermia (43°C) from P14 to P17.

	P14		P15		P16		P17	
	WT	TRPV1^−/−^	WT	TRPV1^−/−^	WT	TRPV1^−/−^	WT	TRPV1^−/−^
N	24	12	24	12	24	12	24	12
Latency (min)	19.91	22.15	14.89	18.02	11.03	13.95	8.35	9.26
Duration (min)	7.42	5.59	10.56	8.36	16.78	12.65	21.37	18.52
Seizure grade	2	2	3	2	4	3	5	4

### Immunohistochemistry

Immature (P18, 20, 22, 24) WT and TRPV1-KO mice were euthanasia after deep anesthesia with isoflurane and successively transcardially perfused with 0.9% saline and 4% paraformaldehyde (PFA). The entire brains were gathered and post-fixed in PFA overnight, and then were kept in 20% and 30% sucrose solution to dehydrate gradually at 4°C until sedimentation. Coronal brain sections were cut into 30 μm thicknesses using a freezing microtome (CM1950, Leica, Germany), and the sections were gathered in a glycerol-based antifreeze solution divided into ten series and mounted on gelatin-coated slides and stored at −20°C until used for immunofluorescence. After being blocked in 5% bovine serum albumin (BSA; Sigma-Aldrich) and 0.1% Triton X-100 (Sigma-Aldrich) in PBS for 1 h at 37°C, tissue sections were incubated overnight at 4°C in 3% BSA containing primary antibodies against the following targets: rabbit anti-TRPV1 (1:250, NB100-98886, Novus), goat anti-Iba1 (1:200, NB100-1028, Novus), mouse anti-CD68/ED1 (1:250, ab31630, Abcam), and mouse anti-toll-like receptor 4 (TLR4; 1:200, sc293072, Santa-Cruz). The sections were washed in PBS (15 min, three times) and incubated with a corresponding secondary antibody [Cy3 goat anti-rabbit (1:500, ab97075, Abcam), FITC donkey anti-goat (1:500, ab6881, Abcam), Alexa 594 goat anti-mouse (1:1,000, ab150120, Abcam), or Dylight 488 goat anti-mouse (1:250, E032210-01, Earthox)] for 1 h at 37°C. After being washed with PBS three times, sections were cover slipped using a glycerol-based mounting medium.

### Immunocytochemistry and Confocal Microscopy

Immunocytochemistry experiments were performed as done for immunohistochemistry. In brief, primary cultured microglia were fixed with 4% PFA and then permeabilized with filtered PBS containing 0.05% Triton X-100 for 15 min. After blocking in 5% BSA and 0.05% Triton X-100 in PBS for 1 h at 37°C, the cells were incubated overnight at 4°C in 3% BSA containing primary antibodies. After washing, the cells were then incubated with the respective secondary antibodies at 37°C. After mounting the coverslips in an anti-fading medium with DAPI, the immunofluorescence images were acquired with a confocal laser-scanning microscope (Leica-LCS-SP8-STED, Leica, Germany).

### Quantification of Microglia and Image Analysis

All quantitative analysis were performed in 3–4 brain slices (near Bregma −2 mm position based on the mouse brain atlas) for all histological analysis as previously described (Zhao et al., 2018). The Iba1 and/or ED1 positive cell counts were performed using a Pannoramic Digital Slide Scanners (3D HISTECH, Ltd.) in the brain cortex. These data are showed as mean value of cells/section, based on average amount of cells in three sections. Furthermore, we sorted a total of 100 Iba1 positive cells in each target zone categorized as ramified, hypertrophic, and amoeboid to analyze the microglial diversely morphological subtypes. The relative quantization of each morphological subtype is represented as the percentage of microglial cells (100 cells/mouse) for each target zone.

### Mouse Primary Culture Cortical Microglia

WT and KO mice (P0) were dissected successively; mice were mercy killed after being sprayed with 75% ethanol, and entire brain placed on a glass garden to isolate microglia. The scalp was separated with scissors, the skull was opened and divided, and the entire brain was stripped and placed in a glass garden pre-cooled dissection medium. Cerebral cortices were separated from the brain and meninges were eliminated. Cortical tissues were dissected and digested in HBSS containing 0.125% trypsin and 0.1% DNase at 37°C for 10 min. Trypsin was replacement with Microglia Complete Media (MCM) and the tissue was washed three times with 4°C dissection medium after trypsin digestion. Dissection tissues were triturated by gentle pipetting and centrifuged in an Eppendorf centrifuge at 1,000 rpm; dissociated cells were seeded on T_75_ flasks in MCM [1× Dulbecco’s modified Eagle’s medium/F12 (DMEM/F12), including 10% heat-inactivated fetal bovine serum (FBS), 1% L-Glutamine, and 1% penicillin/streptomycin mixed solution], then maintained at 37°C in a humidified 5% CO_2_ atmosphere. Experiment was conducted upon cohorts and each cohort consisted of 2–3 mice. Cortices from different mice were mixed together at the step of tissue dissociation. Then the samples were randomly allocated to different treatment groups. After 12 days, the primary cultured mixed glial cells in T_75_ flasks were isolated for 2 h at 200 rpm. The isolated cells were planted in 35-mm dishes (1.0 × 10^6^ cells/dish) or cover glasses (10 mm diameter, 5.0 × 10^4^ cells per glass) for each experiment and used within 3 days.

### Electrophysiology

Whole-cell currents recordings (−60 mV holding potential) were acquired using a MultiClamp 700B and Digidata 1550 (Axon, Molecular Device) at 22–25°C with a pipette made from a Microelectrode (BF150-86-10, Sutter Instruments, Novato, CA, USA) and pulled using a P-2000 puller (Sutter Instruments, Novato, CA, USA). For recording capsaicin responses in cortical microglia, the pipette (tip resistance 4–6 MΩ) was filled with the intracellular solution containing (in mM): 145 KCl, 0.5 EGTA, 2 MgCl_2_, 10 HEPES, and 2 Mg-ATP (pH 7.3 adjusted with KOH, 290 mOsm; Sigma Aldrich). The extracellular and intracellular solution containing (in mM) 145 NaCl, 5 KCl, 1 MgCl_2_, 2 CaCl_2_, 10 HEPES, and 10 D-glucose (pH 7.4 adjusted with NaOH, 310 mOsm). Current-voltage (I/V) relationships were measured using voltage ramps (−60 to +80 mV over 250 ms, 0.5 Hz). Resting membrane potential (RMP) and membrane capacitance were recorded before the perfusion solution. Series resistances (Rs) were monitored throughout the experiment and normally <20 MΩ, and recordings of >20% change in Rs were terminated and discarded. Electrophysiological recorded were filtered at 1.0 kHz and digitized at 50 kHz.

### Live-Cells Calcium Imaging

The protocol for calcium imaging was performed as described (Hassan et al., 2014). The experiments were typically conducted using primary cultured microglia in 5 × 10^5^ cells seeded onto 10 mm glass coverslips coated with PLL. Cultures were washed three times in buffer (145 NaCl, 5 KCl, 2 CaCl_2_, 1 MgCl_2_, 10 HEPES, and 10 Glucose) and loaded for 30 min in the dark at 37°C, with 2 μM Ca^2+^ indicator dye, Fura-2 AM (Beyotime) and 1 μM cell-permeable Pluronic F-127 (Amresco). Cells were then washed three times with HBSS and incubated in extracellular solution at room temperature for 30 min before used in the dark. The intracellular calcium concentration was expressed as the F340/F380 ratios and the signals were captured and analyzed with NIS-Elements AR software (Nikon, Tokyo, Japan). Values were obtained from 150 to 200 cells in time-lapse images from each coverslip. Stimulating drugs were added after 2 min.

### Scratch-Wound Assay

Confluent primary microglia were passaged and seeded onto chambers which being coated with 0.1 mg/ml PLL. After 1 day, a single scratch with a 200 ul sterile pipette tip was made through the microglia monolayer, and cells were washed three times with serum-free media to remove debris and maintained at 37°C in a humidified 5% CO_2_ with MCM. Differential interference contrast (DIC) images of the wounds were acquired at 24 h after scratching using an inverted fluorescence microscope (Nikon, Tokyo, Japan) and NIS-Elements AR software (Nikon, Tokyo, Japan). Each experiment was repeated in triplicate and every well selected randomly three fields to count.

### qPCR

Quantitative polymerase chain reaction (qPCR) was used to verify the relative quantification of RNA (cDNA). Total RNA was extracted by previously isolated mouse brain cortical tissues or primary cultured cortical microglia using Trizol reagent (Invitrogen, Carlsbad, CA, USA), and First-Strand cDNAs was synthesized by reverse transcription with the Revert Aid^TM^ First Strand cDNA Synthesis Kit (Thermo Scientific, Rockford, IL, USA) based on the manufacturer’s specification. qPCR was performed on a SYBR green Real-Time PCR (RT-PCR) master mix kit based on the manufacturer’s specification with primers presented in Table 2. The protocol was performed by the CFX96 sequence detection system (Bio-Rad) was 95°C for 5 min; 40 cycles of 95°C for 15 s, 56°C for 30 s, and 72°C for 20 s. Gene expression for the relative quantification were normalized to the mRNA level of a standard housekeeping gene (*β-actin*) and presented as fold change relative to control using the ΔΔCt method. Melting curve analysis PCR analyses were performed to verify the specificity of the products and each group was repeated at least three independent experiments.

**Table 2:** Primer sequences applied for quantitative polymerase chain reaction (q-PCR).

Primer	Forward prime 5′–3′	Reverse prime 5′–3′	Species
TGF-β1	TAATGGTGGACCGCAACAACG	TCCCGAATGTCTGACGTATTGAAG	Mouse
TβRI	CAAACCACAGAGTAGGCACTAA	CCGATGGATCAGAAGGTACAAG	Mouse
TβRII	GAGTGGCTTCACCACAAAGA	TGTAGGAGGGCAACAACATTAG	Mouse
Arg1	CAACGGGAGGGTAACCATAAG	GAAAGGAACTGCTGGGATACA	Mouse
Chil3	CTGGTGAAGGAAATGCGTAAAG	ATGGTCCTTCCAGTAGGAAATG	Mouse
β-actin	CACGATGGAGGGGCCGGACTCATC	TAAAGACCTCTATGCCAACACAGT	Mouse

### Western Blot Analysis

Western blot was performed based on the manufacturer’s specification. Briefly, isolated brain tissues (100 mg) or primary cultured cells (5 × 10^6^) were resuspended in RIPA lysis buffer (Beyotime) with PMSF. Lysed protein was quantified by BCA protein assay (BCA, Beyotime), separated by SDS-PAGE (10%) and transferred toward PVDF membranes. PVDF blots were then incubated with primary antibodies recognizing rabbit anti-TRPV1 [(1:1,000, NB100-98886, Novus), goat anti-Iba1 (1:500, NB100-1028, Novus), mouse anti-CD68/ED1 (1:1,000, ab31630, Abcam), mouse anti-TLR4 (1:1,000, sc-293072, Santa Cruz, CA, USA), mouse anti-TGF-β1 (1:1,000, MAB240-100, R&D), rat anti-TβR I (1:1,000, MAB5871, R&D), goat anti-TβR II (1:1,000, AF532, R&D), rabbit anti-Arg1 (1:1,000, 9819, CST), goat anti-Ym1 (1:1,000, AF2446, R&D), rabbit anti-GAPDH (1:1,000, ab9485, Abcam), or mouse anti-β-actin (1:100,000, 60008, proteintech)]. After incubation with the corresponding HRP-conjugated secondary antibody, immunoreactive bands were developed by enhanced chemiluminescence (ECL) detection reagent.

### Co-IP

The total protein extracted from the cultured microglia were prepared using the IP lysis buffer, and the concentration of it were detected as described above. For immunoprecipitation analysis, 500–600 μg of the total protein extraction was incubated with 2 μg of rabbit anti-TRPV1 (NB100-1617; Novus), mouse anti-TLR4 (sc-293072, Santa Cruz, CA, USA) or control IgG antibodies and protein A/G agarose beads (Abmart) overnight at 4°C. After being washed three times with 1 ml ice cold IP lysis buffer, immunoprecipitates were boiled and then assessed by western blotting. Three independent experiments were performed.

### Statistical Analysis

**Figure 1:**
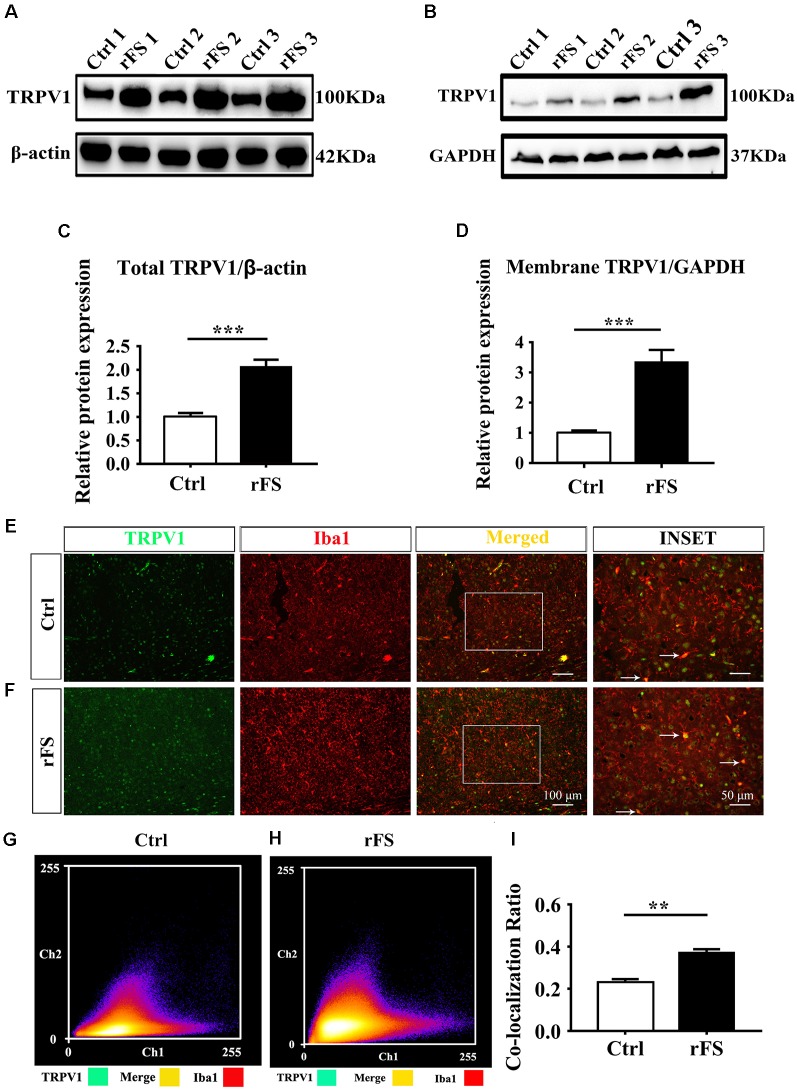
Transient receptor potential vanilloid type 1 (TRPV1) protein expression pattern in cortical microglia after rFS. **(A,B)** Western blot analysis of total protein **(A)** and membrane protein expression **(B)** of TRPV1 in three samples from rFSs group’s mice. **(C,D)** Representative of the quantification analysis of total **(C)** and membrane **(D)** TRPV1 expression were significantly increased in rFSs mice compared with controls (*n* = 6 per groups, ***p < 0.001, unpaired, two-tailed Student’s *t*-test compared with control). **(E,F)** Representative images of immunofluorescence double-labeling for TRPV1 (visualized in green FITC) and Iba1 (visualized in red Alexa Fluor 594) in the cortex after rFS. TRPV1 positive signals highly merged with the Iba1 in the rFS brain (Scale bars: 100 μm). (INSET) White arrow represents the merged panels in magnified images of the squared areas (Scale bars: 50 μm). **(G,H)** Cartogram representation (scatter plot) of the correlation coefficient of Pearson (PCC) for quantifying the co-expression between the TRPV1 and Iba1 in control mice (PCC = 0.22 ± 0.016) and in rFSs mice (PCC = 0.37 ± 0.018). **(I)** The histograms represent the ratio of the mean PCC calculated from the colocalizating in four samples. Data were presented as means ± standard error of the mean (SEM), *n* = 4 per groups, **p < 0.01, unpaired, two-tailed Student’s *t*-test compared with control.

**Figure 2:**
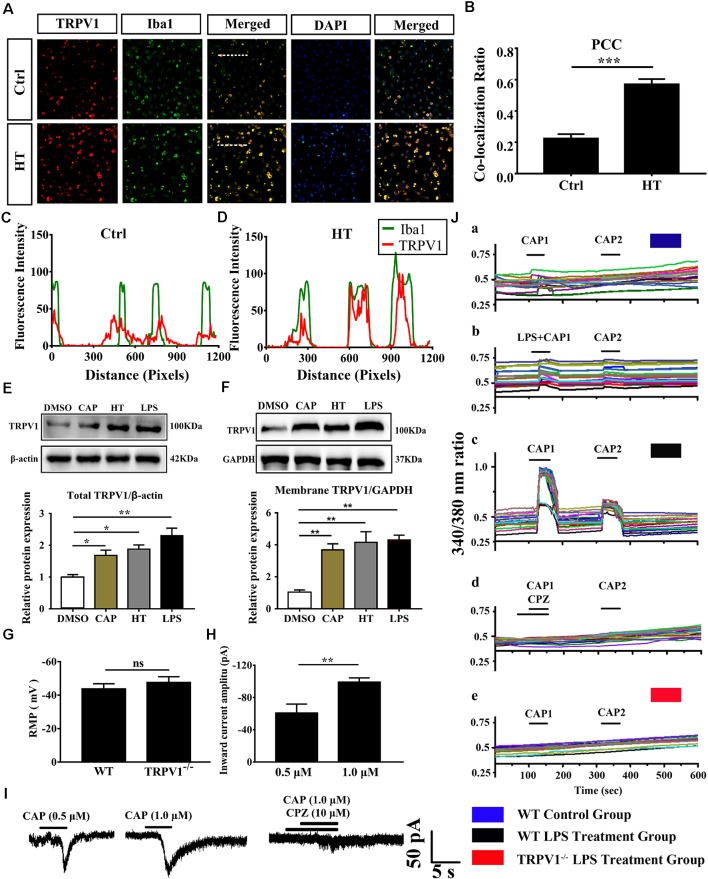
The expressed and electrophysiology properties of TRPV1 in primary cultured microglia.** (A)** Photomicrograph immunocytochemistry indicated different co-expression of TRPV1 (visualized in red Cy3), Iba1 (visualized in green FITC), and cell nuclei (visualized in dark blue DAPI) from control and hyperthermia groups microglia. Note the colocalization indicated by merge yellow fluorescence, the fluorescence intensity of TRPV1 and Iba1 along the indicated line were scanned by using ImageJ software, and their colocalization was determined by the PCC method (**C,D**; Scale bars: 50 μm). **(B)** The histograms represent the ratio of the mean PCC calculated from the colocalizating in three samples (*n* = 3 per groups, ***p < 0.001, unpaired, two-tailed Student’s *t*-test compared with control). **(E,F)** Representative Immunoblot bands and densitometric analysis of total protein **(E)** and membrane protein expression **(F)** of TRPV1 in cortical primary cultured microglia with or without pretreatment of CAP (10 μM), hyperthermia (43°C, 4*30 min) or LPS (1.0 μg/ml, 24 h). Data were presented as means ± SEM, *n* = 3 per groups, *p < 0.05, **p < 0.01, one-way analysis of variance (ANOVA) followed by Dunnett’s multiple comparison test with DMSO. Ctrl, Control; CAP, capsaicin; HT, hyperthermia. **(G)** Whole-cell patch clamp (*I* = 0 mode) recorded resting membrane potential (RMP) in microglia from wild-type (WT) and TRPV1^−/−^ mice (*n* = 11 per groups, ns, not significant, unpaired, two-tailed Student’s *t*-test compared with WT). **(H)** TRPV1 currents amplitude (pA) is significantly increased in 1.0 μM capsaicin group (−99.05 ± 2.632, *n* = 4) than 0.5 μM capsaicin group (−60.73 ± 6.445, *n* = 3) in cortical activated microglia (**p < 0.01, unpaired, two-tailed Student’s *t*-test compared with 0.5 μM CAP). **(I)** Representative whole-cell voltage-clamp mode recorded current traces from individual primary microglia challenged by 0.5 μM, 1.0 μM Capsaicin and Capsazepine (10 μM) applications, the largest current is 104.9 pA in 1.0 μM capsaicin perfusion pretreatment with LPS (1.0 μg/ml, 24 h). **(J)** Representative traces of intracellular calcium ([Ca^2+^]_i_) responses by CAP (1.0 μM), **(Ja,b)** CAP (1.0 μM) with LPS (1.0 μg/ml) in microglia from WT mice. **(Jc,d)** Representative traces of CAP (1.0 μM) induced-([Ca^2+^]_i_) response in LPS (1 ng/ml, 24 h)-stimulated microglia and to Capsazepine (10 μM). **(Je)** Representative traces of CAP (1.0 μM) induced-([Ca^2+^]_i_) response in LPS (1.0 μg/ml, 24 h)-stimulated microglia in TRPV1^−/−^ mice microglia.

**Figure 3:**
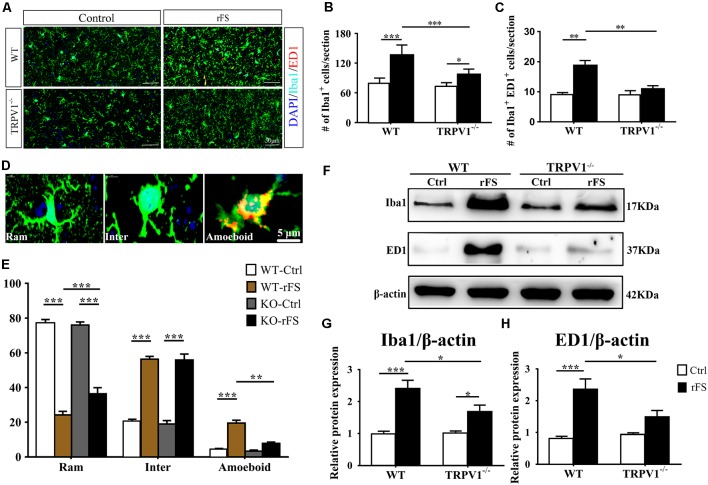
The presence or absence of TRPV1 influences microglial pathophysiological changes in recurrent febrile seizures mice. **(A)** Images of the cortex immunostained for the microglial marker Iba-1 (visualized in green FITC) and the lysosomal marker CD68/ED1 (visualized in red Alexa Fluor 594) and DAPI (visualized in blue) 5 days after rFS from wild type (WT) and TRPV1^−/−^ mice (Scale bars: 50 μm). **(B,C)** Bar graph of quantification of Iba1^+^ **(B)** and Iba1^+^ ED1^+^ **(C)** in 5 days after rFS mice cortex. **(D)** Representative photomicrographs of different microglial morphological subtypes including resting/ramified (Ram), hypertrophic/intermediate (Inter) and round/ameboid (Amoeboid; Scale bars: 5 μm). **(E)** Quantification of relative percentage of microglia with different morphologies in the cortex of WT and TRPV1^−/−^ mice (*n* = 6 per groups, **p < 0.01, ***p < 0.001, two-way ANOVA followed by Tukey’s multiple comparisons). **(F–H)** Representative Immunoblot bands **(F)** and densitometric analysis of Iba1 expression **(G)** and ED1 expression **(H)** were significantly increased in rFS from WT mice than in TRPV1^−/−^ mice. Data were presented as means ± SEM, *n* = 6 per groups, *p < 0.05, **p < 0.01, ***p < 0.001, two-way ANOVA followed by Tukey’s multiple comparisons.

**Figure 4:**
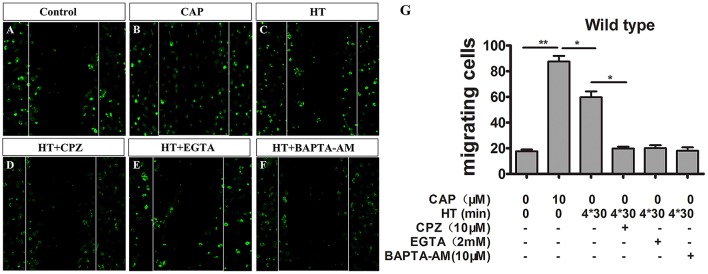
TRPV1 activation enhances migration activity in cultured mice microglia. **(A–F)** Microglial cultures were scratched with tips, and immunocytochemistry was acquired with low magnification at 24 h with or without hyperthermia (43°C) and hyperthermia with CPZ (10 μM) in wild type microglia. **(A)** Control, **(B)** capsaicin (10 μM), **(C)** hyperthermia (4*30 min), **(D)** hyperthermia and capsazepine (CPZ, 10 μM), **(E)** hyperthermia and EGTA (2 mM), **(F)** hyperthermia and BAPTA-AM (10 μM). **(G)** Migration activity of microglia induced by capsaicin (10 μM) and hyperthermia (43°C) with or without Capsazepine (10 μM), EGTA (2 mM), BAPTA-AM (10 μM). Dotted lines show the initial location of the microglia. Notably, the cell number of the microglia invading the wound increases (Scale bar: 100 μm). Data are presented as mean ± SEM, *n* = 3 per groups, *p < 0.05, **p < 0.01, one-way ANOVA followed by Dunnett’s multiple comparison test with control.

**Figure 5:**
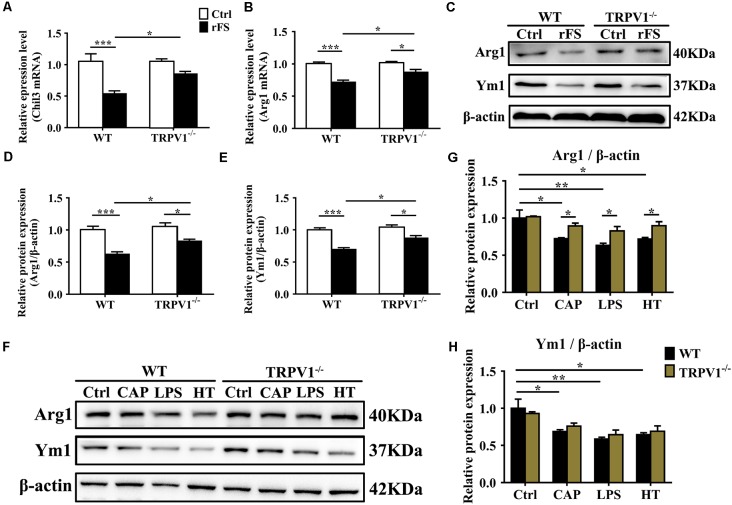
TRPV1 suppresses the expression of Arg1 and Ym1 in activated microglia. **(A,B)** Quantitative PCR (qPCR) analysis of* Chil3* **(A)** and *Arg1* **(B)** mRNA expression in cortical tissues of WT (*n* = 6) and TRPV1^−/−^(*n* = 6) mice after rFS. Values were shown for steady-state transcripts relative to β-actin. Data were presented as mean ± SEM (*n* = 6 per groups, *p < 0.05, **p < 0.01, ***p < 0.001, two-way ANOVA followed by Tukey’s multiple comparisons). **(C–E)** Representative Immunoblot bands **(C)** and densitometric analysis of Arg1 expression **(D)** and Ym1 expression **(E)** were significantly decreased in rFS from WT mice than in TRPV1^−/−^ mice (*n* = 6 per groups, *p < 0.05, ***p < 0.001, two-way ANOVA followed by Tukey’s multiple comparisons). **(F–H)** Representative Immunoblot bands **(F)** and densitometric analysis of Arg1 expression **(G)** and Ym1 expression **(H)** was significantly decreased in the cortex microglia of WT mice than in TRPV1^−/−^ mice after treatment of Capsaicin (10 μM), Hyperthermia (43°C, 4*30 min) and LPS (1.0 μg/ml), respectively. Data were presented as means ± SEM, *n* = 6 per groups, *p < 0.05, **p < 0.01, two-way ANOVA followed by Tukey’s multiple comparisons.

**Figure 6:**
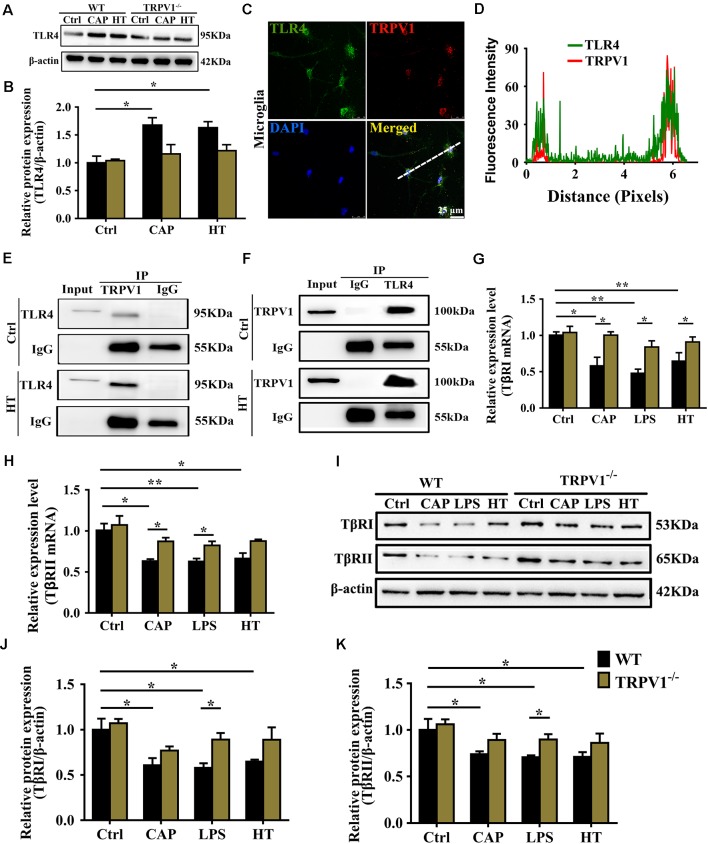
TRPV1 and toll-like receptor 4 (TLR4) mutually inhibited the transforming growth factor-β1 (TGF-β1) signaling in activated microglia. **(A)** Representative western blot bands of TLR4 shows CAP (10 μM) or hyperthermia-induced microglia activation from WT and TRPV1^−/−^ mice. **(B)** Graphic representation protein levels of TLR4 were standardized to β-actin. All data are mean ± SEM (*n* =3, *P < 0.05 vs. control, two-way ANOVA followed by Tukey’s multiple comparisons). **(C)** Photomicrograph immunocytochemistry indicated different co-expression of TRPV1 (visualized in red Cy3), TLR4 (visualized in green DyLight 488), and cell nuclei (visualized in dark blue DAPI) in activated microglia. Note the colocalization indicated by merge yellow fluorescence, the fluorescence intensity of TRPV1 and TLR4 along the indicated line were scanned by using ImageJ software, and their colocalization was determined by the PCC method **(D)**. (Scale bars: 25 μm). **(E,F)** Co-IP showed that interaction between TRPV1 and TLR4 in microglia. Microglia lysates were immunoprecipitated with TRPV1 antibody and then immunoblotted with anti-TLR4 antibody as indicated, vice versa. **(G,H)** qPCR analysis of* TβRI* **(G)** and *TβRII* **(H)** mRNA expression in the cortex microglia of WT mice than in TRPV1^−/−^ mice after treatment of Capsaicin (10 μM), Hyperthermia (43°C, 4*30 min) and LPS (1.0 μg/ml), respectively. Values were shown for steady-state transcripts relative to β-actin. Data were presented as mean ± SEM (*n* = 3 per groups, *p < 0.05, **p < 0.01, two-way ANOVA followed by Tukey’s multiple comparisons). **(I–K)** Representative Immunoblot bands **(I)** and densitometric analysis of TβRI expression **(J)** and TβRII expression **(K)** was significantly decreased in the cortex microglia of WT mice than in TRPV1^−/−^ mice after treatment of Capsaicin (10 μM), Hyperthermia (43°C, 4*30 min) and LPS (1.0 μg/ml), respectively. Data were presented as means ± SEM, *n* = 3 per groups, *p < 0.05, two-way ANOVA followed by Tukey’s multiple comparisons.

All data are expressed as means ± standard error of the mean (SEM) and were analyzed with GraphPad Prism 7 software with appropriate tests for comparisons between WT and TRPV1-KO mice. Student’s *t*-test was used to test the differences between two groups (Figures 1C,D,I, 2B,H,J and Supplementary Figure S1). A one-way analysis of variance (ANOVA) followed by Dunnett’s multiple comparison test were used to analyze the comparisons between multiple groups (Figures 2E,F, 4, and Supplementary Figure S2). A two-way ANOVA followed by Tukey’s multiple comparisons test was used to examine the differences among multiple groups between WT and TRPV1-KO mice (Figures 3, 5, 6). Statistical differences were considered significant at *P* < 0.05. All *P*-values and sample sizes are indicated in the figure legends.

## Results

### Expression and Distribution of TRPV1 in Cortical Microglia of Mice With rFS

To determine the role of TRPV1 in neuroinflammation-induced seizures, we first investigated the expression and distribution of TRPV1 in the mouse cortex after rFS (Figure 1). One day after rFSs, the expressed TRPV1 was immunodetected as a dominant band of about 100 kDa in the brain tissue of total and membrane protein extracts from WT mice (Figures 1A,B). Western blot analyses showed a significant up-regulation of TRPV1 total protein in the cortex tissue of rFS mice compared with controls (Figures 1A,C). Importantly, the levels of TRPV1 membrane protein increased significantly in rFS mice cortex tissue compared with controls (Figures 1B,D). To further verify the specific pattern of TRPV1 expression in cortical microglia of mice with rFS, we examined the expression of TRPV1 and Iba1 (a specific marker of microglia) through double-immunostaining in rFS mice. One day after rFS, TRPV1 was detected to be expressed in some of the microglia, although not all microglia were TRPV1-embedded in controls and rFS mice (Figures 1E,F). When dual immunofluorescence of TRPV1 and Iba1, colocalization was observed in acute brain slices. Compared with the weak immunoreactivity and weak degree of colocalization with TRPV1 and Iba1 by the Pearson coefficient of correlation (PCC) in mouse cortex of the controls (PCC = 0.23 ± 0.016, *n* = 4; Figure 1G), a moderate degree of colocalization with TRPV1 and Iba1 immunoreactivity was observed in the cortex of rFS mice (PCC = 0.37 ± 0.018, *n* = 4; Figure 1H). As shown in Figure 1I, histograms represent the ratio of the mean PCC using Image J software. The Pearson correlation values range from −1 to +1. A correlation of 1 indicates complete colocalization and a correlation of 0 indicates no colocalization, and a correlation of −1 indicates a negative interaction between the two proteins. These findings indicate that the amount of colocalization between TRPV1 and Iba1 was weak in control mice, but the PCC for TRPV1 and Iba1 immunoreactive colocalization was moderate in rFS mice.

Growing evidence suggests that TRPV1 is unevenly expressed in microglia and is primarily localized in intracellular endomembrane system rather than on the plasma membrane (Miyake et al., 2015). To determine whether TRPV1 expression and distribution have changed in cultured cortical microglia after being activated, we further validated the expression of TRPV1 through immunocytochemistry and revealed that the TRPV1 channel exhibited a punctate cytoplasmic and plasma membrane distribution in activated microglia from WT mice (Figure 2A). Presumably, TRPV1 immunostaining was observed in ~70% of microglia (Figure 2A). We calculated the PCC to quantify the degree of their colocalization; the rate of colocalization was much higher between TRPV1 and Iba1 in microglia with hyperthermia treatment (PCC = 0.224 ± 0.016) than control microglia (PCC = 0.57 ± 0.019, *p* = 0.0002; Figure 2B). Furthermore, we observed colocalization pixels for TRPV1 and Iba1 to be present on microglia, and TRPV1 strongly co-localizes with Iba1 in microglia with hyperthermia treatment (Figures 2C,D). Thus, these PCCs were higher, showing colocalization between TRPV1 and Iba1 in microglia with hyperthermia treatment than controls.

Consistent with the dominant colocalization between TRPV1 and Iba1, we found that TRPV1 was highly expressed in activated cultured microglia compared with that in controls (Figure 2E). Similarly, the membrane TRPV1 expression of microglia with hyperthermia, LPS or capsaicin treatment was significantly increased over that of control microglia (Figure 2F). Interestingly, the TRPV1 expression in microglia with hyperthermia was higher than that in microglia treated with the TRPV1 agonist capsaicin, suggesting that hyperthermia, as a combined injury element, could modulate the TRPV1 expression and trafficking through multiple intracellular mechanisms (Figures 2E,F).

Taken together, these findings suggest that TRPV1 expresses in the microglia of the mouse cortex and increases the total and membrane protein expression in the rFS brain and activated microglia.

### Electrophysiological Properties of TRPV1 in Mice Primary Cultured Microglia

Based on the above findings, we performed whole-cell patch-clamp recording of primary cultured cortical microglia to directly detect the functional properties of TRPV1 in different states of cultured microglia. Consistent with the canonical electrophysiological properties of TRPV1 channels in microglia (Miyake et al., 2015; Marrone et al., 2017), RMP of the recorded microglia did not significantly differ in WT mice and TRPV1^−/−^ mice (WT: −43 ± 3, *n* = 11, TRPV1^−/−^: −47 ± 3; *n* = 11; *p* > 0.05; Figure 2G). Unexpectedly, no typical inward current was induced by capsaicin (1.0 μM) in resting microglia (*n* = 12) at the holding potential of −60 mV. Once activated, the capsaicin-evoked current density was enlarged to 104.9 pA (*n* = 7) after LPS (1.0 μg/ml, 24 h) stimulation. The capsaicin-induced inward currents in a concentration-dependent manner in microglia, since the current amplitude increased gradually after pretreatment with 1.0 μg/ml LPS for 24 h, from group −60.73 ± 6.445 pA (*n* = 3) in 0.5 μM capsaicin to −99.05 ± 2.632 pA (*n* = 4) in 1.0 μM capsaicin (Figures 2H,I). All currents evoked by capsaicin in microglia should be TRPV1 currents, since 10 μM capsazepine, a specific antagonist of TRPV1, had an inhibitory effect on the current evoked by capsaicin in LPS (1.0 μg/ml, 24 h)-pretreated microglia, with a suppression ratio of 90% (Figure 2I).

To further address whether the TRPV1 channel is functionally expressed in microglia and whether capsaicin can evoke TRPV1 currents through elevation of the intracellular Ca^2+^ concentration in activated microglia, we verified the effect of capsaicin on the microglial intracellular Ca^2+^ concentration ([Ca^2+^]_i_) using the high-affinity Ca^2+^ indicator Fura-2 AM. Microglia showed no capsaicin- or LPS-evoked calcium influx in the resting state (Figure 2Ja,b). Once the microglia were activated, capsaicin (1.0 μM) evoked an increase of the [Ca^2+^]_i_ in LPS-stimulating (1.0 μg/ml, 24 h) microglia from WT mice, but this effect was significantly inhibited in capsazepine (10 μM) pretreatment (Figure 2Jc,d). However, no response to capsaicin was detected in microglia from TRPV1^−/−^ mice (Figure 2Je). Live-cells Calcium imaging revealed calcium permeability of the TRPV1 increase in activated microglia (Figure 2J).

Altogether, these findings indicate that TRPV1 is functionally expressed in microglia and their electrophysiological activities enhance remarkably in activated states.

### TRPV1 Activation Aggravates the Microglial Pathophysiological Changes in the Cortex After rFSs

Microglia play important roles in immune surveillance and brain homeostasis in the healthy brain, and shift to an polarized state that is characterized by significant changes in morphology and function during seizure (Hu et al., 2015; Eyo et al., 2017). We investigated whether stimulation of TRPV1 regulates the activation of microglia in the brain after rFS (Figure 3, and Supplementary Figure S1). Coronal sections were immunostained with anti-Iba1, the marker for all microglia (green), and with anti-ED1, the marker for activated microglia (Zhao et al., 2018; red: also named CD68) at 1, 3, 5 and 7 days after rFSs (Supplementary Figure S1A).

Morphometric analyses showed profound microglial proliferation and polarization in the cortex, with increased entire microglia amounts observed as well as the presence of polarized microglial phenotypes from 1 to 7 days after rFSs, especially at 5 days following rFSs from WT mice (Supplementary Figures S1B,C) and TRPV1^−/−^ mice (Supplementary Figures S1B,D). Therefore, we selected the 5-day mice to analyze the morphological characteristics. The amount of Iba1 positive microglial cells was increased in the cortex from WT and TRPV1^−/−^ mice with rFS compared to that in control mice, but the number of Iba1^+^ cells was significantly reduced in TRPV1^−/−^ mice (Figures 3A,B). Similarly, activated microglial cells, quantified as the numbers of Iba1^+^ and ED1^+^ double-positive cells, were increased in the cortex in rFS compared to control mice, whereas TRPV1^−/−^ mice expressed lower amounts of ED1^+^ cells compared with that in WT mice with rFS (Figure 3C). These data suggest that activated TRPV1 channel promotes the proliferation of cortical microglia.

In addition, the ramified morphology of microglia facilitates their contact with neurons and is therefore critical for central nervous system (CNS) homeostasis (Nimmerjahn et al., 2005; Zhao et al., 2018). Accordingly, we further magnified the images and assessed the morphological phenotypes of microglia; the shape of microglia changed canonically from ramified to amoeboid, and the expression of Iba1 and ED1 in cortex indicated that the amoeboid microglia had an activated phenotype (Figure 3D). Morphometric analyses showed that the majority of microglia from nonhyperthermia treatment in acute brain slices had a ramified morphology, manifested as smaller cell bodies with thin and elongated branches (Figures 3D,E). Compared with nonhyperthermal controls, we observed a mass of hypertrophied Iba1 positive microglia cells displaying larger cell bodies and thicker, shorter and highly branched processes with less branching in rFS mouse brain slices; furthermore, the percentage of resting/ramified microglia was reduced by approximately 80% along with an increased hypertrophied and amoeboid morphology compared to control mice, which suggested a more polarized phenotype in the cortex and hippocampus (Figures 3D,E). Of note, microglial morphological changes were significantly alleviated in brain sections from TRPV1^−/−^ mice after rFS (Figure 3E). Thus, microglia adopt a reactive-like morphology in the cortex brain when the TRPV1 channel is activated.

Based on these findings, we further corroborated the function of TRPV1 in microglial polarization using western blot analysis of protein extracts of cortex tissues (Figure 3F). The protein levels of Iba1 and ED1 were significantly increased in rFS mice compared with the levels in control mice, whereas TRPV1^−/−^ rFS mice expressed lower protein levels of Iba1 and ED1 than WT rFS mice did (Figures 3G,H). These results suggest that loss of TRPV1 function prevented excessive microglial activation *via* hyperthermic stimulation.

### Activation of TRPV1 Promoted the Migration of Microglia

Cell migration is a canonical manifestation of the microglia polarization. Recent studies indicate capsaicin-mediated, concentration-dependent migration in microglia (Miyake et al., 2015), yet no studies have examined whether TRPV1 activation regulates microglial migration through hyperthermic stimulation. Therefore, to investigate thoroughly the physiopathological role of TRPV1 with respect to hyperthermia in microglia, we tested whether hyperthermia affected microglial migration by performing scratch-wound models. Stimulation with hyperthermia for 24 h or capsaicin (1 μM) promoted microglia migration toward the cell-free area, which was remarkably suppressed by co-treatment with capsazepine (10 μM; Figures 4A–D,G). By contrast, we observed that pre-treatment with capsaicin or hyperthermia did not affect the microglial migration activity in TRPV1^−/−^ mice (Supplementary Figure S2). Since Ca^2+^ is a key regulator of cell motility and mainly cation through the TRPV1 channel (Miyake et al., 2015), we synchronously applied hyperthermia with EGTA (2 μM) or BAPTA-AM (10 μM, an intracellular Ca^2+^ chelator) to explore the mechanism underlying TRPV1-induced migration in microglia. The EGTA and BAPTA-AM markedly inhibited the hyperthermia-induced migration in microglia derived from WT mice (Figures 4E–G). By contrast, neither EGTA nor BAPTA-AM affected microglia migration derived from TRPV1-KO mice (Supplementary Figure S2). These results confirm that hyperthermia activates TRPV1 and subsequently promotes microglial migration.

### TRPV1 Activation Suppresses Alternative Microglial Activation

Microglia are considered as resident macrophages, mediating the innate immune response and releasing pro-/anti-inflammatory mediators when confronted with endogenous or exogenous stimuli (Zhao et al., 2018), and Arginase 1 (Arg1, *Arg1*) and Chitinase 3-like 3 (Ym1, *Chil3*) are known to be downstream effectors in TGF-β1-induced alternative microglial activation (Raes et al., 2005; Benson et al., 2015). Accordingly, we investigated the effects of TRPV1 knockout on the regulation of Arg1 and Ym1 during rFS. qPCR analysis showed that *Arg1* and *Chil3* significantly decreased within 5 days after rFS (Figures 5A,B). Similarly, Arg1 and Ym1 protein expression significantly decreased in WT mice compared with TRPV1^−/−^ mice with hyperthermia-induced seizures relative to their respective controls (Figures 5C–E). Conversely, the gene and protein expression levels of Arg1 and Ym1 were increased in TRPV1^−/−^ mice compared with WT mice (Figures 5A–E). These data demonstrate that TRPV1 is likely to govern the regulation of Arg1 and Ym1 during hyperthermia-induction seizures.

To further explore the influence of TRPV1 on TGF-β1-induced microglia-alternative activation, we performed, respectively, treatment with capsaicin (1 μM), hyperthermia and LPS (1 μg/ml) for 24 h in primary microglia. Since the morphological change could not always accurately reflect the polarization states, the evaluation of the alternative activation is still dependent on molecular markers such as Arg1 and Ym1. Therefore, we analyzed the expression levels of Arg1 and Ym1 in activated microglia. Consistent with the change in tissue, the enhanced expression of Arg1 and Ym1 protein levels by TRPV1 activation with an agonist or hyperthermia was reduced in both capsaicin-stimulated microglia and LPS-stimulated microglia (Figures 5F–H). Interestingly, microglial-specific expression of M2 markers (Arg1 and Ym1) have a similar variation trend in both LPS-stimulated and capsaicin-treated microglia, suggesting a coherent effect between LPS and TRPV1.

### Interaction of TRPV1 With TLR4 and Inhibited the TGF-β1 Signaling in Activated Microglia

Next, we continued to investigate the molecular mechanism underlying the decrease in Arg1 and Ym1 driven by microglial TRPV1 activation. TLR4 opposes the actions of TGF-β1 and inhibits the Arg1 and Ym1 expression to ensure sustaining microglial activation that eventually may contribute to neuroinflammatory diseases (Mitchell et al., 2014). Furthermore, TLR4 signaling activation can enhance the function of TRPV1 *via* intracellular signaling (Assas et al., 2014; Min et al., 2014; Li et al., 2015). Hence, we speculate that TRPV1 stimulation of microglia may inhibit the release of Arg1 and Ym1 through the synergistic effect between TRPV1 and TLR4.

To confirm the hypothesis that TRPV1 binds to TLR4, contributing to seizures by inhibiting the effect of TGF-β1, we first confirmed that activation of TRPV1 either by capsaicin or hyperthermia markedly enhanced the expression of TLR4 in the WT microglia (Figures 6A,B). However, no significant changes in TLR4 protein were observed in TRPV1-KO microglia (Figures 6A,B). Then, colocalization experiments were conducted using specific TLR4 and TRPV1 antibodies. Confocal analysis of dual immunofluorescence showed that TLR4 and TRPV1 are colocalized in microglia (Figure 6C). TRPV1-positive microglia are shown in red, and TLR4 positive microglia are shown in green, and the yellow color shows the merged images of microglia positive for both TRPV1 and TLR4 (Figure 6C). Furthermore, we observe colocalization pixels for TRPV1 and TLR4 to be present on microglia, and TRPV1 strongly co-localizes with TLR4 in microglia (Figure 6D).

Co-IP assays were performed by identifying the interaction between TRPV1 and TLR4 (Figures 6E,F). The Co-IP experiments indicated that TRPV1 was able to precipitate TLR4 and vice versa, suggesting that TRPV1 and TLR4 are directly associated with each other (Figures 6E,F). These results suggest that TLR4 was more highly expressed in activated microglia, allowing TRPV1 to bind to it. This interaction will amplify TLR4 signaling *via* antagonizing the TGF-β1 downstream anti-inflammatory process.

We next determined the effects of TRPV1 activation on TGF-β1 signaling in microglia. Conforming to the microglial electrophysiological properties, the capsaicin current and calcium influx following microglial activation by LPS suggest a synergistic effect between LPS and capsaicin. Capsaicin (1 μM), LPS or hyperthermia treatment resulted in a significant reduction in TGF-β receptor I (TβRI) and TGF-β receptor II (TβRII) mRNA at 24 h (Figures 6G,H). Western blot analysis also demonstrated reduced TβRI and TβRII after capsaicin, LPS or hyperthermia treatment (Figures 6I–K). By contrast, TβRI and TβRII gene and protein expression showed no difference in activated TRPV1-deficient microglia, indicating that TRPV1 may inhibit TGF-βR to attenuate the anti-inflammatory response of microglia (Figures 6I–K).

Together, our results suggest that microglial TRPV1 activation suppresses the expression of genes/proteins known to be significantly involved in TGF-β1 signaling through an interaction with TLR4.

## Discussion

In this study, we uncovered the following four findings: first, TRPV1 not only was functionally expressed in the microglia but also showed increased expression in rFS mice as well as in hyperthermia- or LPS-induced reactive microglia, especially with respect to the membrane expression of TRPV1. Second, TRPV1 currents and capsaicin-induced intracellular Ca^2+^ influx was observed in LPS-stimulated reactive microglia. Third, TRPV1 activation triggers marked proliferation, migration and morphology changes. Fourth, TRPV1 activation suppressed the microglial M2 markers (Arg1 and Ym1) *via* interaction with TLR4. These findings provide the first direct evidence that microglial TRPV1 may be an important trigger in the progression of seizures.

Although there is widespread acknowledgment that the mechanism of seizures is generally assumed to be an imbalance between excitatory and inhibitory neurotransmission, the therapeutic strategies targeting these mechanisms are insufficient for a radical clinical cure (Eyo et al., 2017). However, neuroinflammation is a novel focus of current research (Colonna and Butovsky, 2017; Eyo et al., 2017). As brain-resident macrophages, microglia reside nearly uniformly throughout the entire brain and play a pivotal role in maintaining brain microenvironment (Colonna and Butovsky, 2017; Eyo et al., 2017). Reactive microglia are the initiators of neuroinflammation and are widely involved in the development of various neurological diseases, including epilepsy (Robel and Sontheimer, 2016; Colonna and Butovsky, 2017; Marrone et al., 2017). Recently, growing clinical and experimental evidence has suggested that microglial activation is a characteristic of epileptic brains, and this activation mainly manifests as microglial phenotypic and morphological changes, including proliferation, migration and induced abundant pro-inflammatory and/or anti-inflammatory biomarkers (Aronica and Crino, 2011; Boche et al., 2013; Eyo et al., 2017; Zhao et al., 2018). Therefore, how are microglia activated to mediate neuroinflammation during seizure generation?

Many researches have demonstrated the essential function of TRPV1 in seizures at the brain level (Kong et al., 2014; Jia et al., 2015; Saffarzadeh et al., 2015, 2016). Our previous studies suggest that mRNA and protein expression of TRPV1 were significantly increased in rFS mice rather than in the control mice (Huang et al., 2015). Clinical study suggests that TRPV1 is expressed in higher amounts in mTLE patients (Sun et al., 2013). TRPV1 is functionally expressed in a subpopulation of microglia and is primarily localized in intracellular organelles rather than on the plasma membrane of microglia under physiological conditions (Miyake et al., 2015; Marrone et al., 2017). In this work, we provide the first report of high levels (especially, surface expression) of TRPV1 in rFS mice and activated microglia, which suggests that microglial TRPV1 may rapidly facilitate surface trafficking from intracellular organelles in response to certain stimuli (Kong et al., 2017). Therefore, TRPV1 may serve as an evaluation biomarker for seizure generation.

As a novel detector and biomarker of neuroinflammation (Marrone et al., 2017), TRPV1 plays a pivotal role in neuroinflammation-induced seizures, by regulating the microglia-neuron communication through promoting neuroinflammation, disrupting brain homeostasis, and increasing excitatory neurotransmission release (Eyo et al., 2017).

The microglial M1 phenotype is the main pathway by which TRPV1 activation promotes microglia activation and neuroinflammation-induced seizures. There is considerable evidence indicating that microglial M1 markers are overexpressed in epileptic foci and cerebrospinal fluid (CSF; Aronica and Crino, 2011; Benson et al., 2015; Eyo et al., 2017; Kong et al., 2017) and that TRPV1 increases excitatory neurotransmission by promoting the pro-inflammatory response and the microvesicular or ectosomal shedding of microglia (Naziroğlu and Övey, 2015; Saffarzadeh et al., 2016; Marrone et al., 2017). We demonstrated that TRPV1 promotes cortical microglial proliferation, activation and migration after rFS and contributes to seizures by suppressing microglial M2 polarization. This is reminiscent of a previous finding that activation of TRPV1 could directly affect the microglial pathophysiological response and shift microglia to a amoeboid-like morphology and M1 polarization state by increasing the production and release of pro-inflammatory mediators, including TNF, IL-1β, IL-6, HMGB1 and ROS (Schilling and Eder, 2009; Hassan et al., 2014; Huang et al., 2015; Miyake et al., 2015). Microglia treatment with capsaicin produced remarkably higher levels of TNF and lower levels of IL-10 derived from WT mice, whereas TRPV1^−/−^ microglia express an equal number of TNF and remarkably greater amounts of IL-10 compared with WT cells (Marrone et al., 2017), consistent with the results of our previous research on BV2 (Huang et al., 2015). Thus, the activation of TRPV1 induces a microglial M1 polarization from WT mice; conversely, microglia shift toward a M2 polarization derived from TRPV1^−/−^ mice (Marrone et al., 2017).

In addition, TRPV1 inhibits the microglial M2 phenotype, which is also a complementary pathway in epileptogenesis. Some studies have already confirmed that M2 markers are upregulated or downregulated during epileptogenesis (Xanthos and Sandkuhler, 2014; Yu et al., 2014; Benson et al., 2015; Ali et al., 2017), and insufficient Arg1 or Ym1 is a susceptibility element for epileptogenesis (Boche et al., 2013; Benson et al., 2015; Cantero et al., 2016). One recent study reported that microglial-specific expression levels of M2 markers (Arg1, Ym1) were significantly downregulated at the early chronic phase in a pilocarpine-induced epilepsy model (Benson et al., 2015). We found that Arg1 and Ym1 were consistently decreased in the rFS brain and activated microglia. However, Arg1 and Ym1 maintained the normal level when TRPV1 was deficient, suggesting that the microglia shifted from the M1 to the M2 phenotype. As previously reported, TRPV1-KO prevented the pro-inflammatory effects of LPS by maintaining microglial M2 polarization state (Marrone et al., 2017); we also observed that activation of TRPV1 inhibited the expression of Arg1 and Ym1 in primary cultured microglia, whereas TRPV1-KO attenuated the suppression effect of hyperthermia or LPS. These results suggest that, as a complementary mechanism for neuroinflammation-induced seizures, the TRPV1-inhibited microglial M2 phenotype results from unbalance of the microglial M1/M2 phenotype.

As already mentioned, we found that increased expression of TRPV1 was characteristic of the LPS-induced microglial M1 phenotype. By whole-cell patch-clamp and live-cells calcium-imaging experiments, we further determined the electrophysiological characteristics of TRPV1 in primary cultured mouse microglia. Consistent with previous studies (Miyake et al., 2015), we could not record the typical TRPV1-like currents and calcium influx in resting microglia by capsaicin. However, the application of capsaicin to LPS-stimulated (1.0 μg/ml, 24 h) microglia elicited both inward current and elevated intracellular calcium concentration, which could be inhibited by TRPV1 antagonist capsazepine. This finding suggests that TRPV1 is functionally expressed in activated microglia and plays a pivotal role in LPS-stimulated microglial polarization. Thus, we hypothesized that TRPV1 interacts with TLR4 involvement in microglia-mediated neuroinflammation. To verify the hypothesis, classical LPS-stimulated microglial M1 polarization state was used as a positive control. Consistent with LPS treatment, activation of TRPV1 with capsaicin significantly increased the expression of IL-1β, IL-6, TNF and HMGB1 in BV2, while TRPV1-KO markedly reduced these inflammatory mediators (Huang et al., 2015). Recent clinical specimen analysis showed that the inflammatory mediators (TLR4, IL-1β and IL-10) were overexpressed in CSF and brain tissues of mTLE with hippocampal sclerosis patients (Leal et al., 2017). The deficiency and/or blockade of TRPV1 prevents the pro-inflammatory effects of LPS by maintaining microglial M2 polarization state (Marrone et al., 2017). Consistent with the TRPV1, TLR4 activation increases the susceptibility of rodents to febrile seizures and increases seizure-induced pro-inflammatory cytokine and microglial polarization (Eun et al., 2015; Wang et al., 2018). All of these studies suggest that TRPV1 appears to work synergistically with TLR4 to potentiate seizures and exacerbate seizure-induced inflammatory responses.

Indeed, a direct functional interaction between TLR4 and TRPV1 has been shown in HEK293 cells and in both rodent and human spinal cord slices, and this interaction plays an important role in numerous immune-mediated disorders (Assas et al., 2014; Min et al., 2014; Li et al., 2015). In this study, we also observed TLR4/TRPV1-coexpression and interaction in microglia of seizures. TLR4 is a critical factor of microglia M1 activation; once activated, TLR4 decreases the expression of the TGF-β1 receptors (TβRI and TβRII) and Smad2/3, a pivotal mediator of TGF-β1 signaling (Mitchell et al., 2014). Consistently, we also found that activation of TRPV1 could inhibit the microglial TGF-β1 receptors (TβRI and TβRII), thereby inhibiting Arg1 and Ym1 expression and promoting seizures. LPS also antagonizes the effect of TGF-β1 and inhibits its related cytokines.

**Figure 7:**
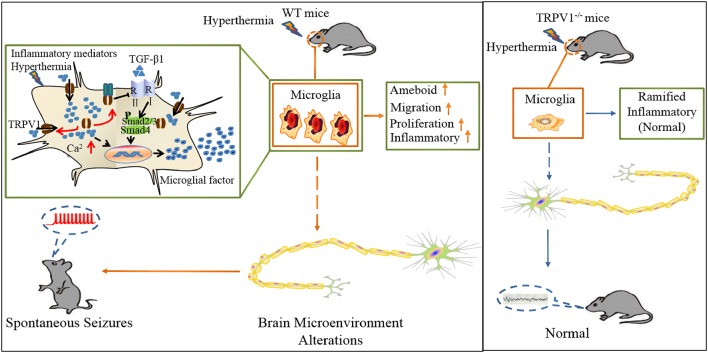
Schematic representation of TRPV1 in inhibiting the microglial TGF-β1 signaling *via* TLR4 after hyperthermia-induction seizures. (1) TRPV1 is upregulated and trafficked to the membrane from microglia; (2) TRPV1 leads to excessive Ca^2+^ influx in microglia and promotes microglia polarization; (3) TRPV1 activation triggers marked proliferation, migration, morphology changes and inflammatory responses; and (4) increased microglial TRPV1 inhibited TGF-β1 signaling and Arg1 and Ym1 *via* interaction with TLR4. Consistently, TRPV1^−/−^ have the microglial M2 phenotype.

In summary, we delineate an intriguing molecular mechanism underlying TRPV1-inhibited microglial TGF-β1 signaling *via* interaction with TLR4 after hyperthermia-induction seizures (Figure 7). Acute brain injury such as hyperthermia can cause TRPV1 and microglial activation, which is the prominent feature in neuroinflammation. Reactive microglia express a high level of TRPV1, which can be activated by intracellular inflammatory mediators and trafficked to the membrane. Activation of TRPV1 is coupled to the TLR4 signaling pathway and subsequent suppression of TGF-β1 signaling in microglia, which in turn aggravates microglial M1 activation after rFSs. This situation leads to excessive microglial activation and the release of pro-inflammatory mediators, thus resulting in epileptiform activities and the development of seizures. Our results suggest a vital role for TRPV1 in neuroinflammation after rFSs and that TRPV1 can potentially serve as a therapeutic target for the management of seizures.

## Data Availability Statement

All raw data used in this manuscript are available from the corresponding author on reasonable request.

## Ethics Statement

This study was carried out in accordance with the recommendations of “Institutional Animal Care and Use Committee of Wuhan University Medical School.” The protocol was approved by the “Institutional Animal Care and Use Committee of Wuhan University Medical School.”

## Author Contributions

WK, WH and BP conceived and designed the experiments. WK, XW and XY performed the experiments. WK and XW analyzed the data. SH, JY, WL and WH contributed to the reagents, materials and analysis tools. WK and BP wrote the article. All authors reviewed and approved the final manuscript.

## Conflict of Interest

The authors declare that the research was conducted in the absence of any commercial or financial relationships that could be construed as a potential conflict of interest.

## Supplementary Material

FIGURE S1 Microglia rapid proliferation in cortex from WT mice after rFS. **(A)** Timeline of the experimental protocol for tracing microglial proliferation after rFS from WT mice and TRPV1^−/−^ mice. **(B)** Representative images of the cortex labeled with DAPI and Iba1 in control and rFS mice (Scale bar: 50 μm). **(C,D)** Quantification of Iba1-positive microglia in WT and TRPV1^−/−^ mice. Average values represent the mean ± SEM, *n* = 3 per groups, ***p* < 0.01, ****p* < 0.001 vs. control, two-way ANOVA Tukey’s multiple comparisons test.

FIGURE S2 TRPV1^−/−^ inhibit microglial migration. **(A–F)** Microglial cultures were scratched with tips, and phase-contrast images were acquired with low magnification at 24 h with or without hyperthermia (43°C) and hyperthermia with CPZ (10 μM) in TRPV1^−/−^ microglia. **(A)** Control, **(B)** capsaicin (10 μM), **(C)** hyperthermia (4*30 min), **(D)** hyperthermia and capsazepine (CPZ, 10 μM), **(E)** hyperthermia and EGTA (2 mM), **(F)** hyperthermia and BAPTA-AM (10 μM). **(G)** Migration activity of microglia induced by capsaicin (10 μM) and hyperthermia (43°C) with or without Capsazepine (10 μM), EGTA (2 mM), BAPTA-AM (10 μM). Dotted lines represent the initial position of the microglia. Note that the number of microglia invading the wound is increased (Scale bar: 100 μm). Data were presented as means ± SEM, *n* = 3 per groups, one way ANOVA followed by Dunnett’s multiple comparison test with control.
