# Supplementary figures and images for: Activation of TRPV1 Contributes to Recurrent Febrile Seizures via Inhibiting the Microglial M2 Phenotype in the Immature Brain
Source: Front Cell Neurosci. 2019 Oct 11;13:442. doi: 10.3389/fncel.2019.00442 (PMC6798794; doi:10.3389/fncel.2019.00442)

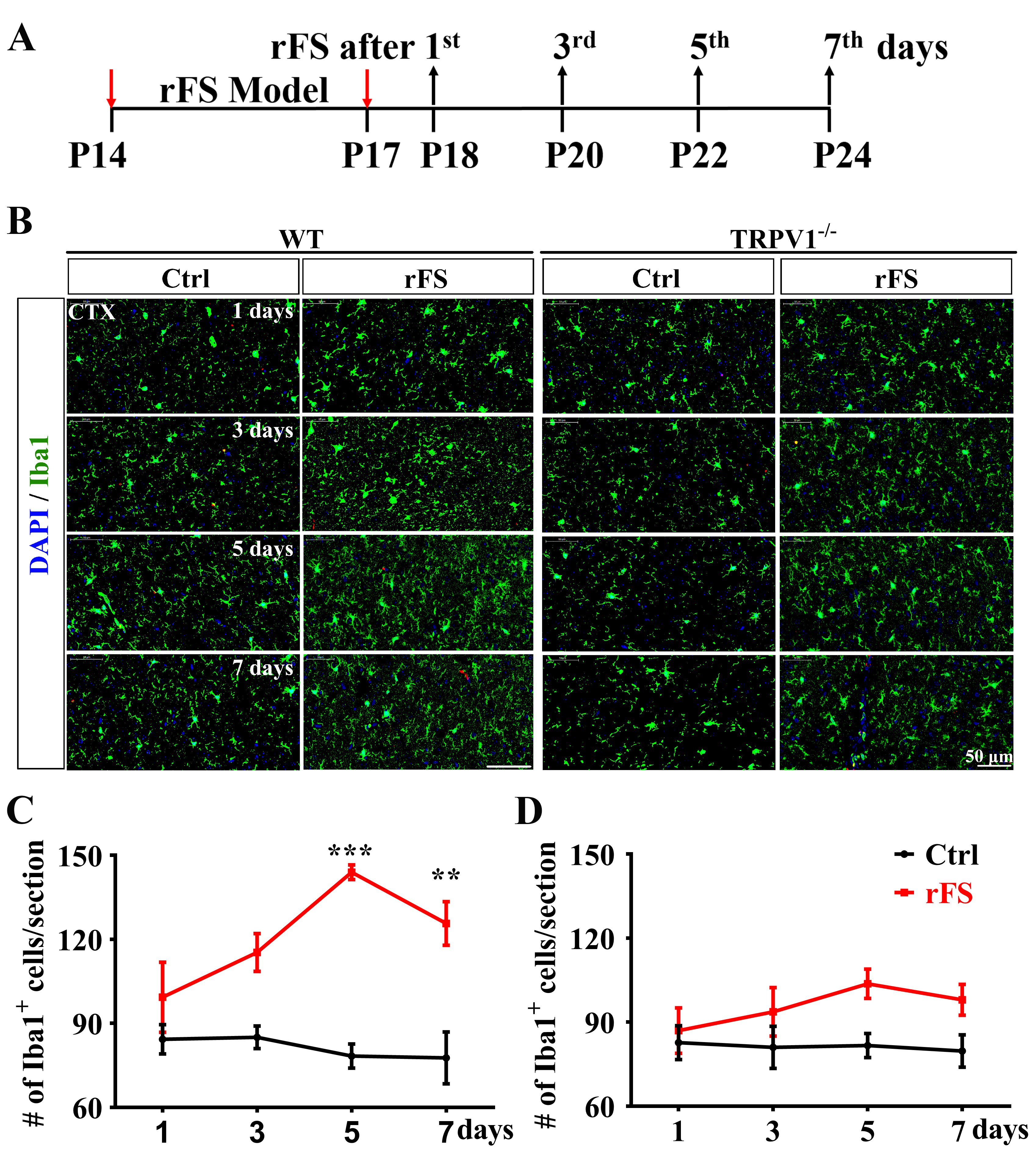

Supplement: FIGURE S1 — Microglia rapid proliferation in cortex from WT mice after rFS. (A) Timeline of the experimental protocol for tracing microglial proliferation after rFS from WT mice and TRPV1−/− mice. (B) Representative images of the cortex labeled with DAPI and Iba1 in control and rFS mice (Scale bar: 50 μm). (C,D) Quantification of Iba1-positive microglia in WT and TRPV1−/− mice. Average values represent the mean ± SEM, n = 3 per groups, **p < 0.01, ***p < 0.001 vs. control, two-way ANOVA Tukey’s multiple comparisons test. [file Image_1.TIF]

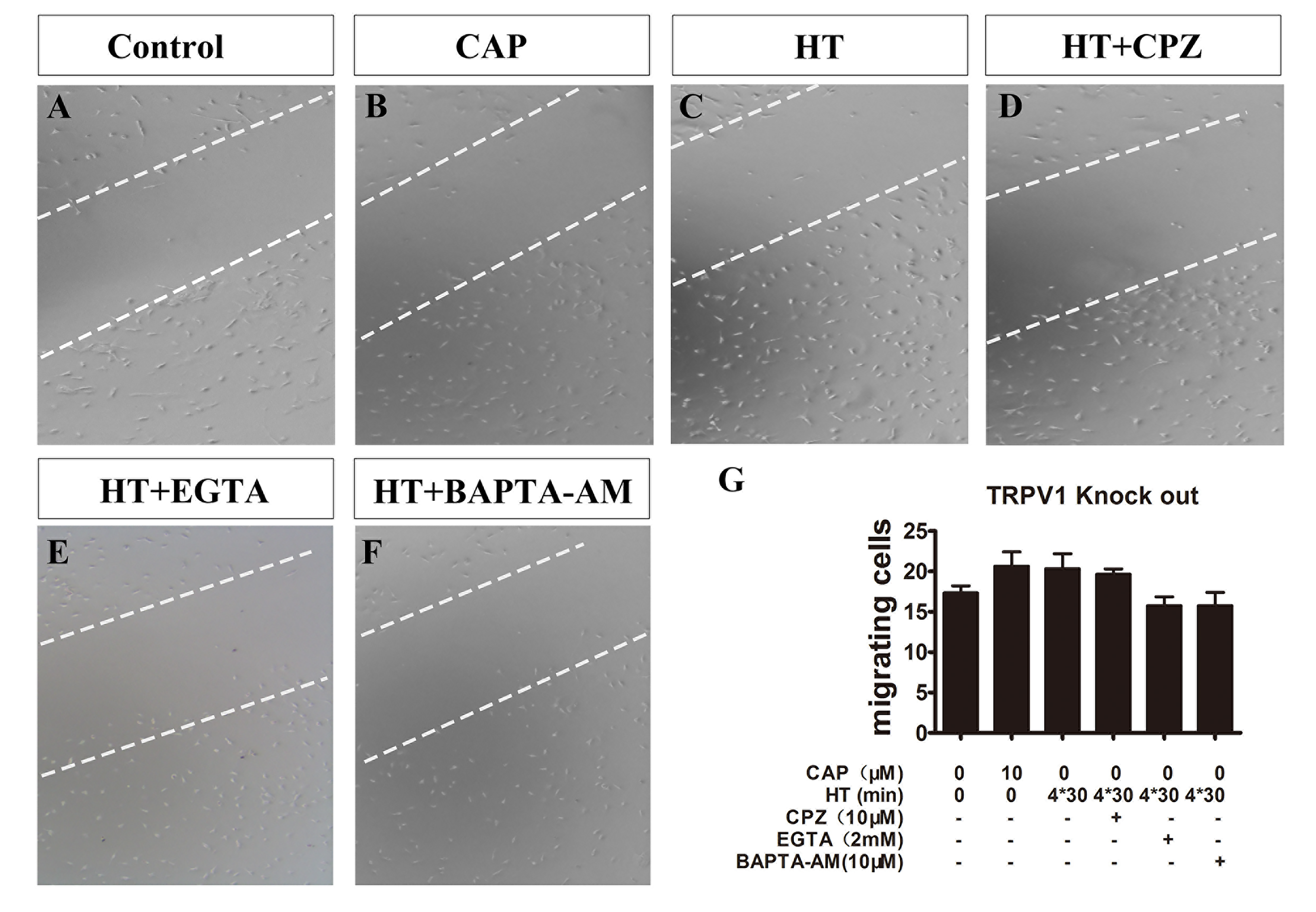

Supplement: FIGURE S2 — TRPV1−/− inhibit microglial migration. (A–F) Microglial cultures were scratched with tips, and phase-contrast images were acquired with low magnification at 24 h with or without hyperthermia (43°C) and hyperthermia with CPZ (10 μM) in TRPV1−/− microglia. (A) Control, (B) capsaicin (10 μM), (C) hyperthermia (4*30 min), (D) hyperthermia and capsazepine (CPZ, 10 μM), (E) hyperthermia and EGTA (2 mM), (F) hyperthermia and BAPTA-AM (10 μM). (G) Migration activity of microglia induced by capsaicin (10 μM) and hyperthermia (43°C) with or without Capsazepine (10 μM), EGTA (2 mM), BAPTA-AM (10 μM). Dotted lines represent the initial position of the microglia. Note that the number of microglia invading the wound is increased (Scale bar: 100 μm). Data were presented as means ± SEM, n = 3 per groups, one way ANOVA followed by Dunnett’s multiple comparison test with control. [file Image_2.TIF]
